# Supplementary material for: Electrochemical surface passivation of LiCoO2 particles at ultrahigh voltage and its applications in lithium-based batteries
Source: Nat Commun. 2018 Nov 21;9:4918. doi: 10.1038/s41467-018-07296-6 (PMC6249257; doi:10.1038/s41467-018-07296-6)
Supplement: Supplementary file 1 — Supplementary Information [file 41467_2018_7296_MOESM1_ESM.pdf]

Supplementary Information

Electrochemical surface passivation of $LiCoO_2$ at ultrahigh voltage and its applications in lithium-based batteries

Qian et al.

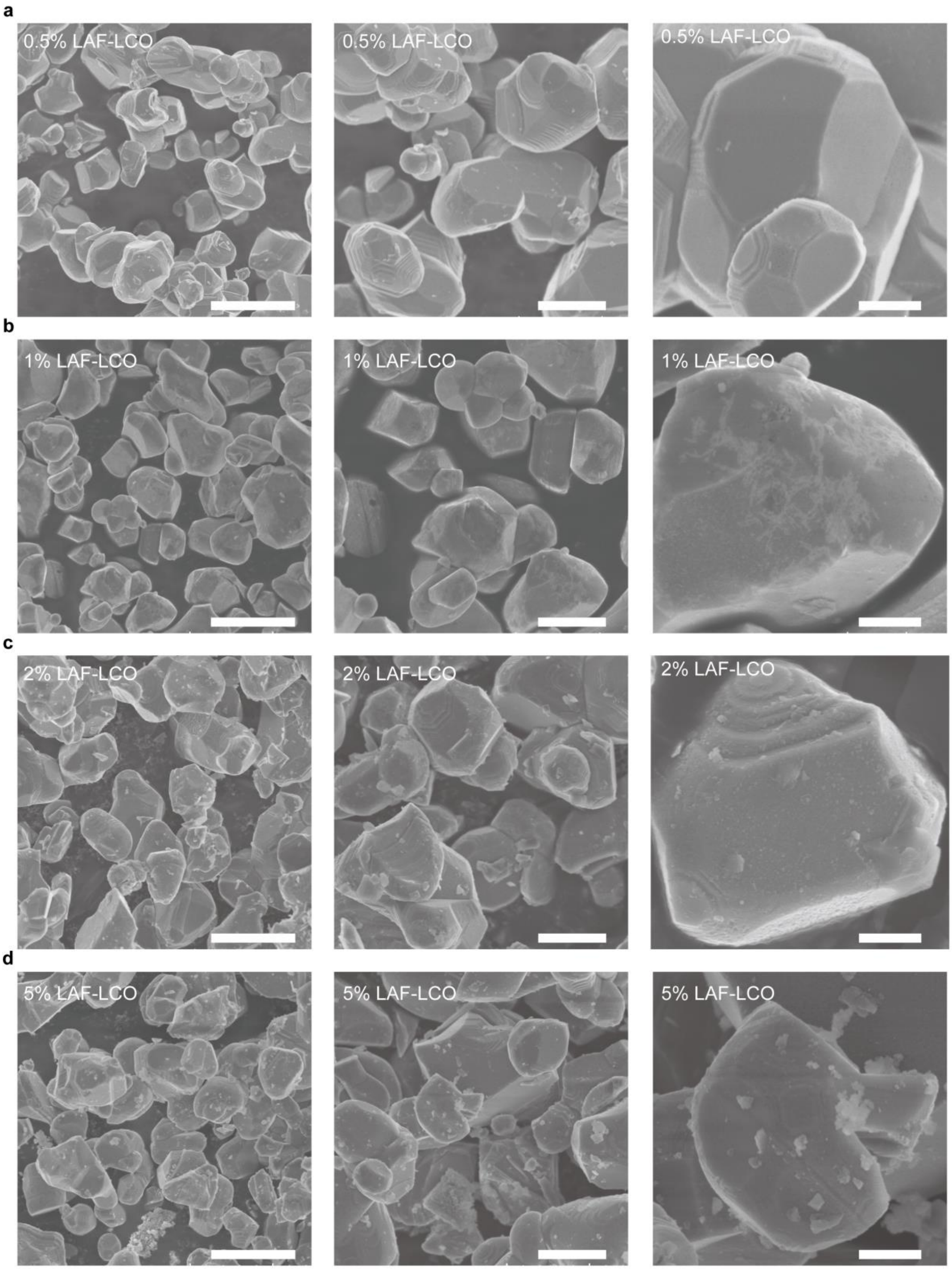

a
0.5% LAF-LCO
0.5% LAF-LCO
0.5% LAF-LCO
b
1% LAF-LCO
1% LAF-LCO
1% LAF-LCO
c
2% LAF-LCO
2% LAF-LCO
2% LAF-LCO
d
5% LAF-LCO
5% LAF-LCO
5% LAF-LCO

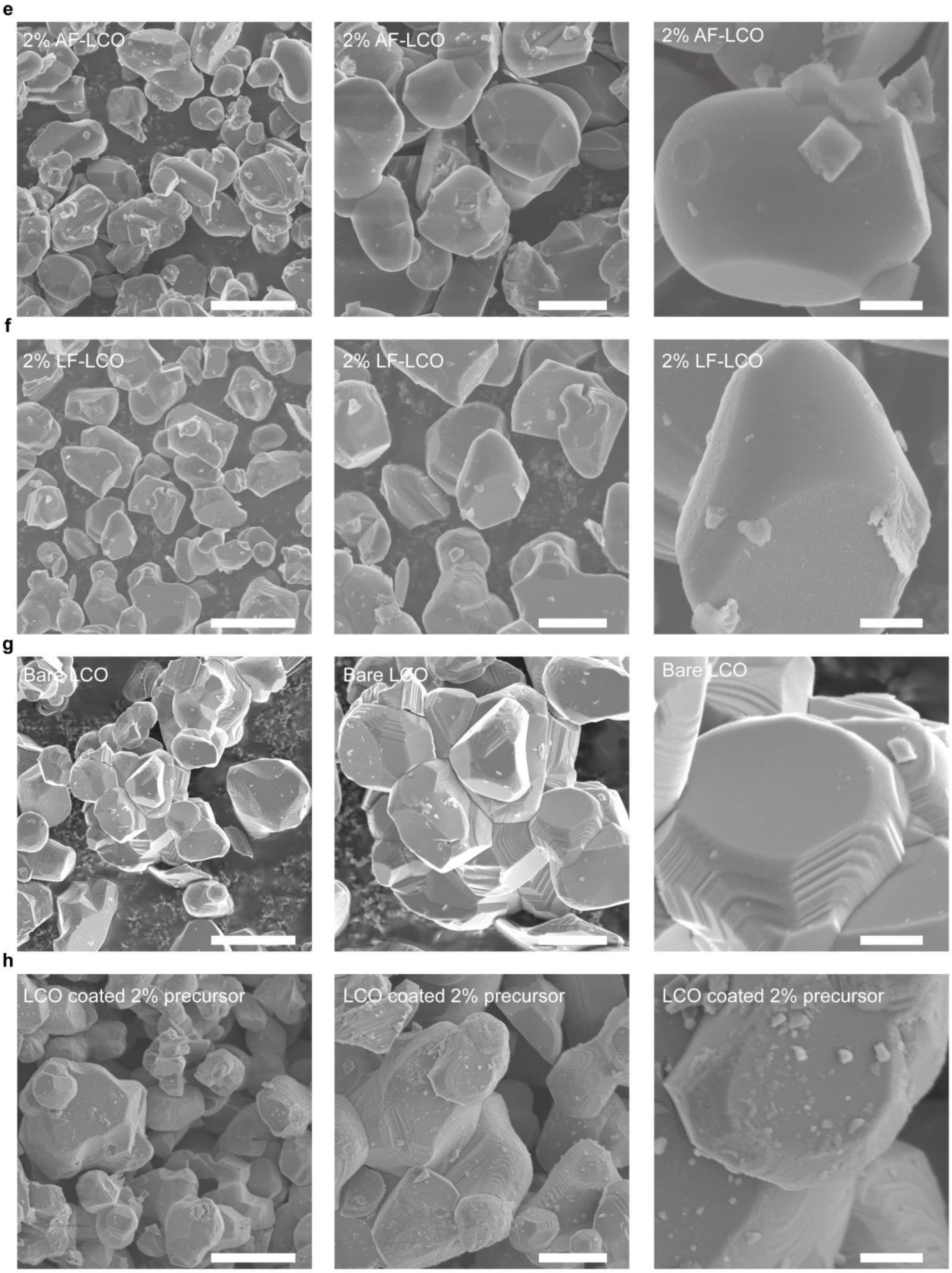

Supplementary Figure 1 | Morphologies of LAF/AF/LF/bare LCO. The SEM images of **a** 0.5% LAF-LCO. **b** 1% LAF-LCO. **c** 2% LAF-LCO. **d** 5% LAF-LCO. **e** 2% AF-LCO. **f** 2% LF-LCO. **g** bare LCO. **h** LCO coated with 2% precursor after hydrothermal process. Scale bar = 5 μm, 2 μm, 500 nm, respectively. Scale bar: 5 μm, 2 μm, 500 nm, respectively.

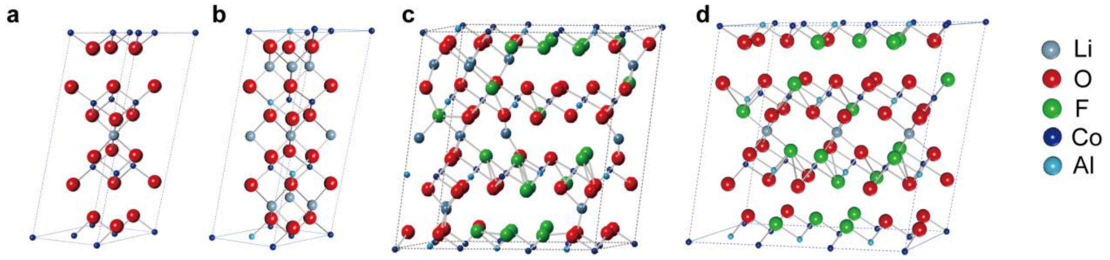

Supplementary Figure 2 | Atomic structures of a $Li_{1/9}CoO_2$, **b** $LiAl_{1/3}Co_{2/3}O_2$, **c** $Li_{1/3}Al_{1/3}Co_{2/3}O_{4/3}F_{2/3}$, **d**, $L_{1/9}iAl_{1/3}Co_{2/3}O_{4/3}F_{2/3}$.

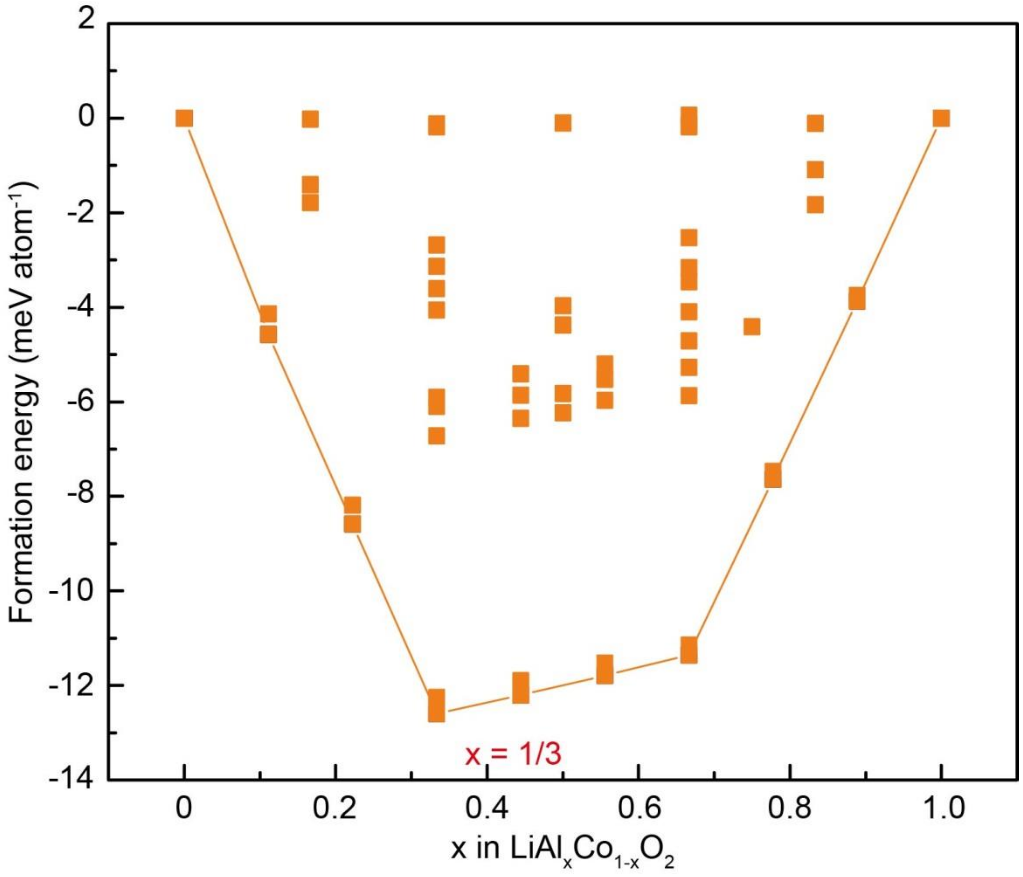

Supplementary Figure 3 | Formation energy of $LiAl_xCo_{1-x}O_2$ at different concentrations x. The energy is calculated with reference to the energies of $LiAlO_2$ and $LiCoO_2$.

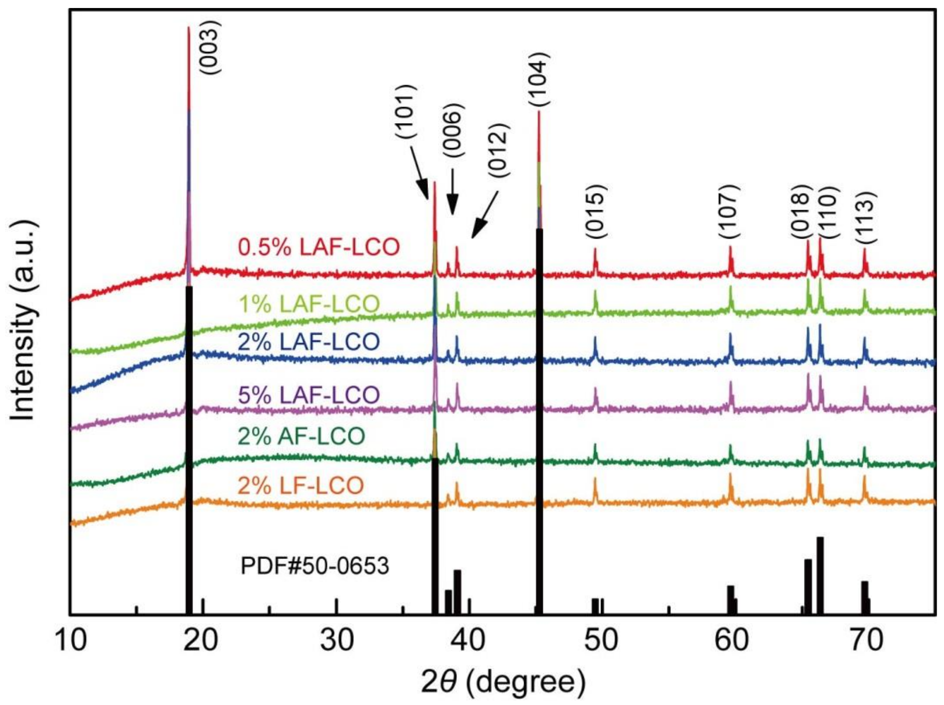

Supplementary Figure 4 | XRD patterns of bare LCO (standard PDF#50-0653) and LAF/AF/LF-LCO. Source data are provided as a Source Data file.

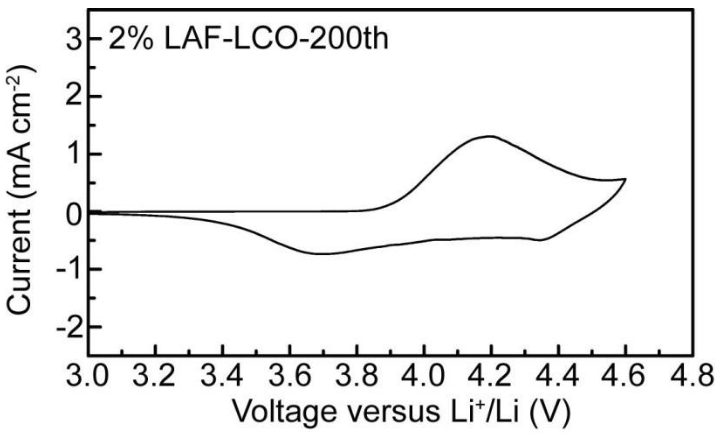

Supplementary Figure 5 | Cyclic voltammogram of cells with 2% LAF-LCO electrodes at a scan rate of 0.1 mV s^{-1} in the voltage range of 3.0-4.6 V (vs. Li^+/Li) after 200 cycles. Source data are provided as a Source Data file.

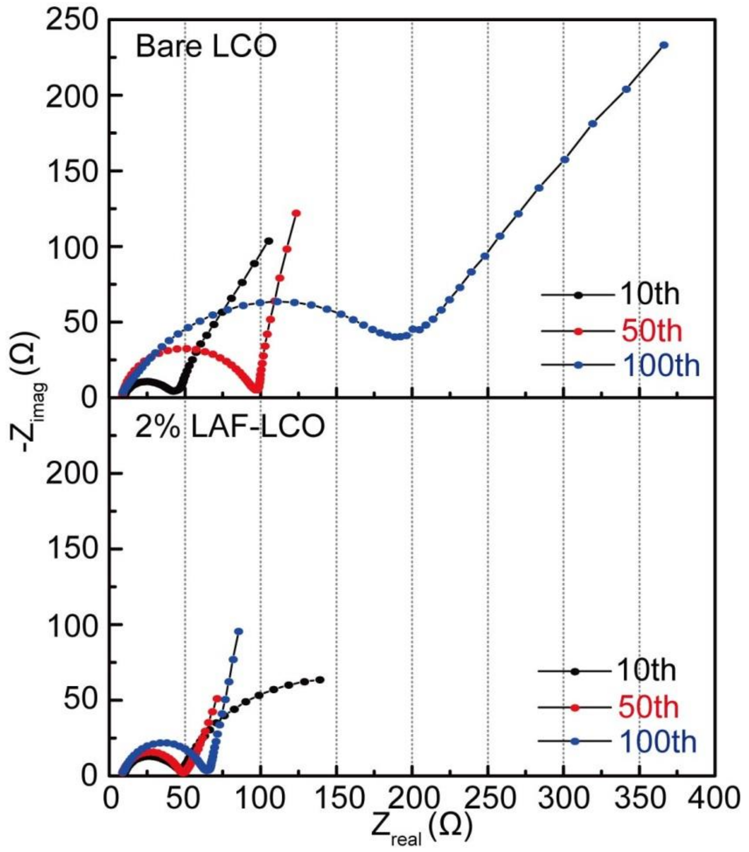

Supplementary Figure 6 | Electrochemical impedance spectra of cells with bare LCO or 2% LAF-LCO electrodes. Source data are provided as a Source Data file.

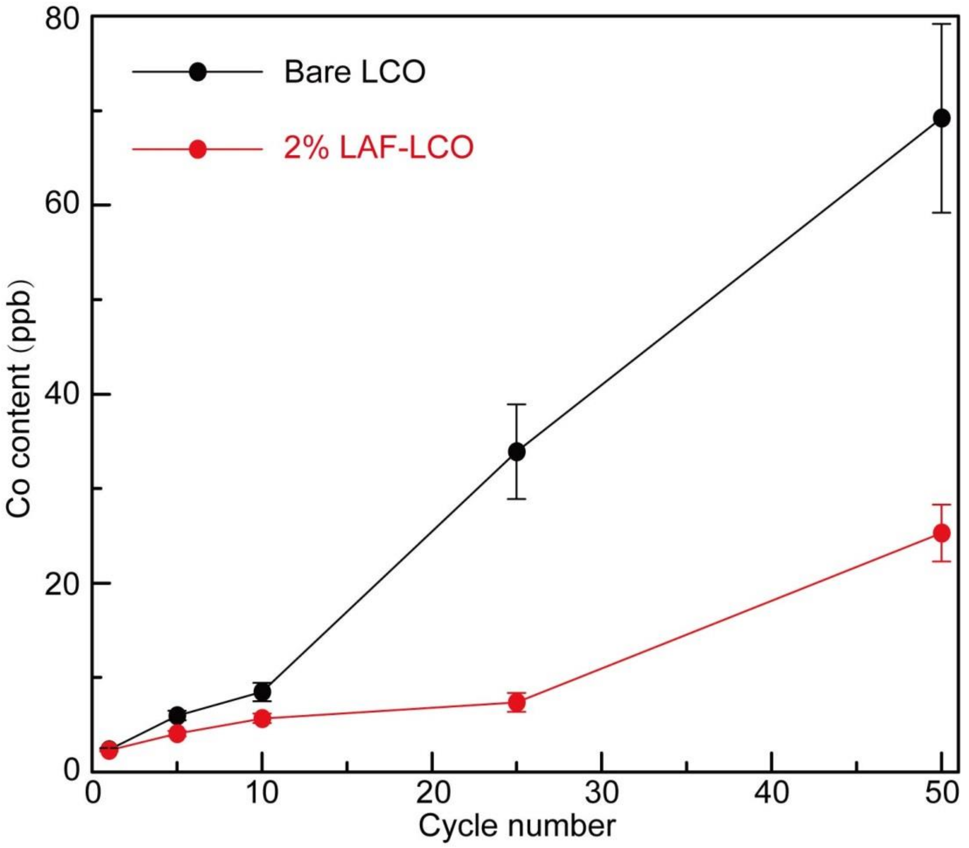

Supplementary Figure 7 | Co content dissolved in the electrolyte of EC:DEC (v:v = 1:1) at different cycles, corresponding to the cells in Fig. 4b. Source data are provided as a Source Data file.

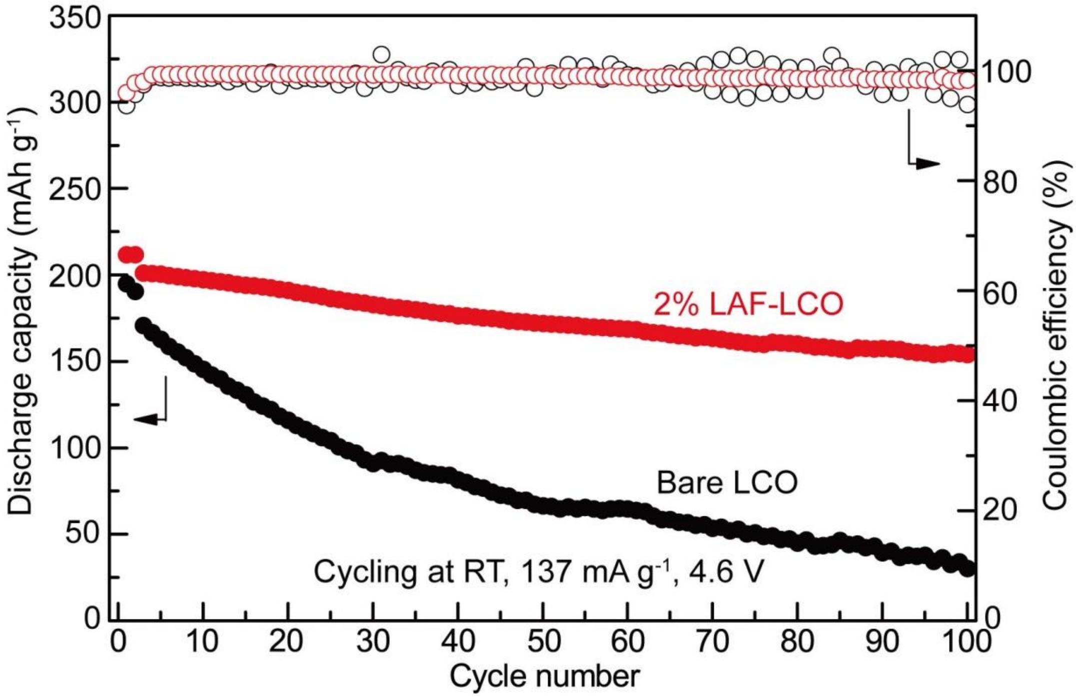

Supplementary Figure 8 | Cycling performance of the cells with bare LCO or 2% LAF-LCO electrodes at room temperature in the voltage range of 3.0-4.6 V (vs. Li^+/Li) at current density of 137 mA g^{-1}. Source data are provided as a Source Data file.

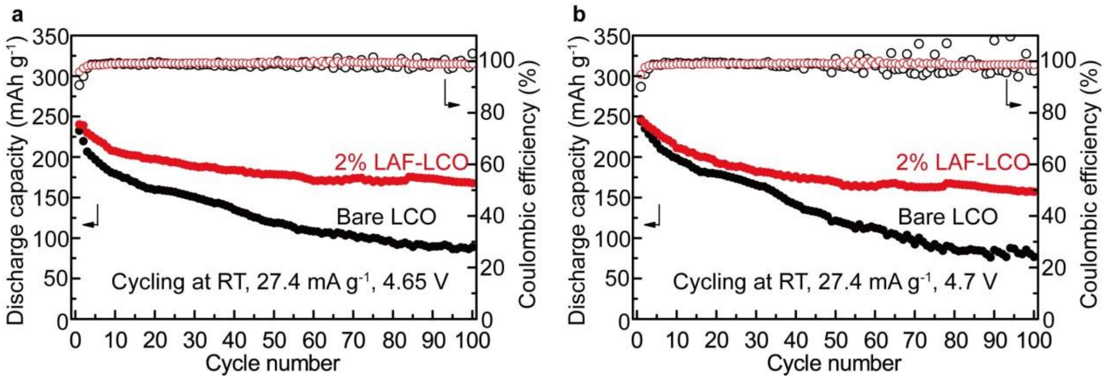

Supplementary Figure 9 | Cycling performance of the cells with bare LCO or 2% LAF-LCO electrodes at room temperature in the voltage range of 3.0-4.65 V or 3.0-4.7 V (vs. Li^+/Li) at current density of 27.4 mA g^{-1}, respectively. Source data are provided as a Source Data file.

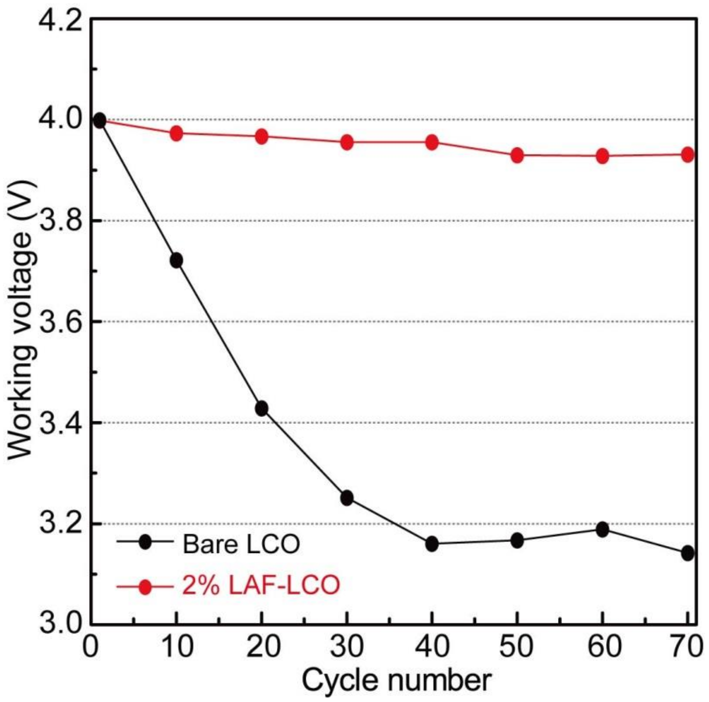

Supplementary Figure 10 | Working voltages of half-cells with 2% LAF-LCO electrodes or bare LCO electrodes at different cycles corresponding to Fig. 4b. Source data are provided as a Source Data file.

a

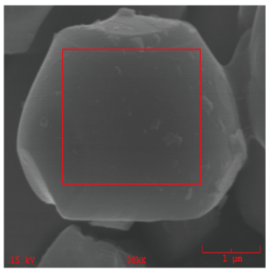

b

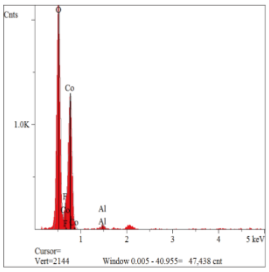

c

Elt.	Line	Intensity (c/s)	Atomic %	Atomic Ratio	Conc	Units	Error 2-sig	
O	Ka	609.18	68.083	1.0000	38.098	wt.%	0.581	
F	Ka	17.29	2.094	0.0308	1.386	wt.%	0.213	
Al	Ka	14.47	0.851	0.0125	0.799	wt.%	0.155	
Co	Ka	278.28	28.972	0.4255	59.717	wt.%	1.360	
			100.000		100.000	wt.%		Total

Supplementary Figure 11 | The elemental amount from EDX measurement.

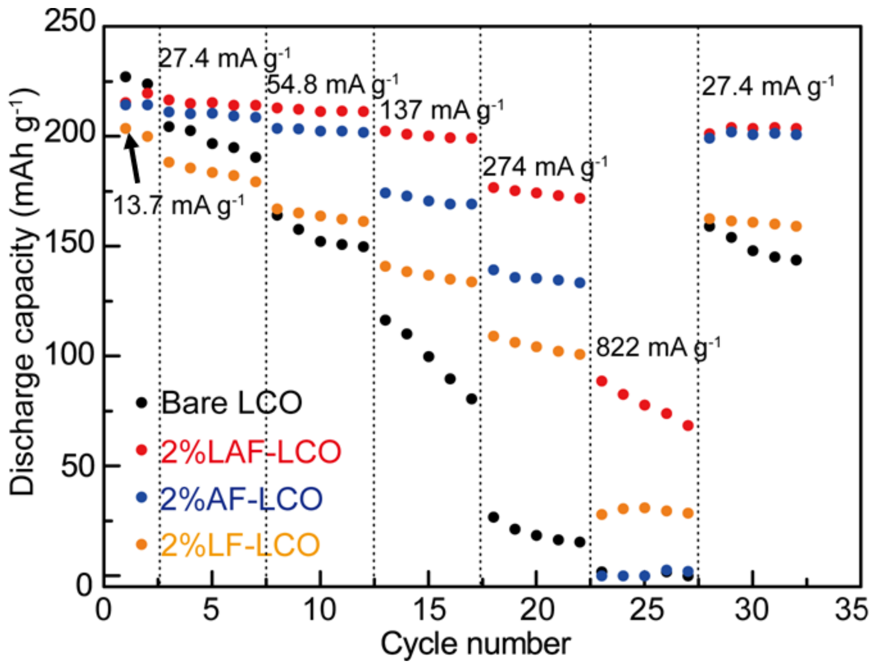

Supplementary Figure 12 | Rate performance of half-cells with bare LCO and 2% LAF/AF/LF -LCO electrodes at room temperature in the voltage range of 3.0-4.6 V (vs Li^+/Li). All cells were pre-cycled for 2 cycles at low current density of 13.7 mA g^{-1}. Source data are provided as a Source Data file.

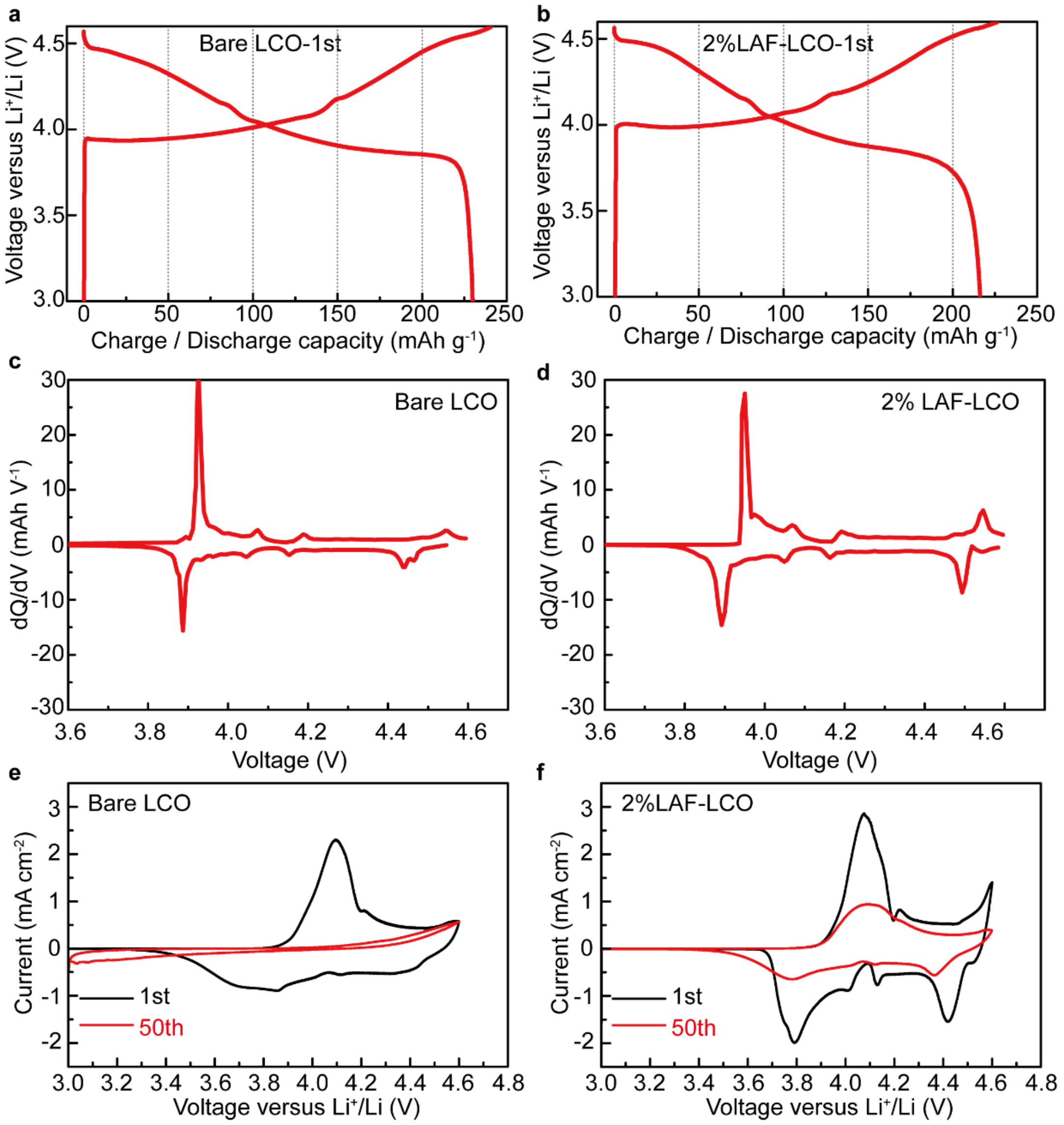

Supplementary Figure 13 | The analyses for phase transition of bare LCO and 2% LAF-LCO. a,**b** Discharge-charge profiles of half-cells with bare LCO or 2% LAF-LCO electrodes at 1st cycle in the voltage range of 3.0-4.6 V (vs. Li^+/Li) at current density of 27.4 mA g^{-1}. **c**,**d** The dQ/dV curves of cells with bare LCO or 2% LAF-LCO electrodes corresponding Supplementary Fig. 11. a and b, respectively. **e**,**f** Cyclic voltammograms of cells with bare LCO or 2% LAF-LCO electrodes at a scan rate of 0.1 mV s^{-1} in the voltage range of 3.0-4.6 V (vs. Li^+/Li). Source data are provided as a Source Data file.

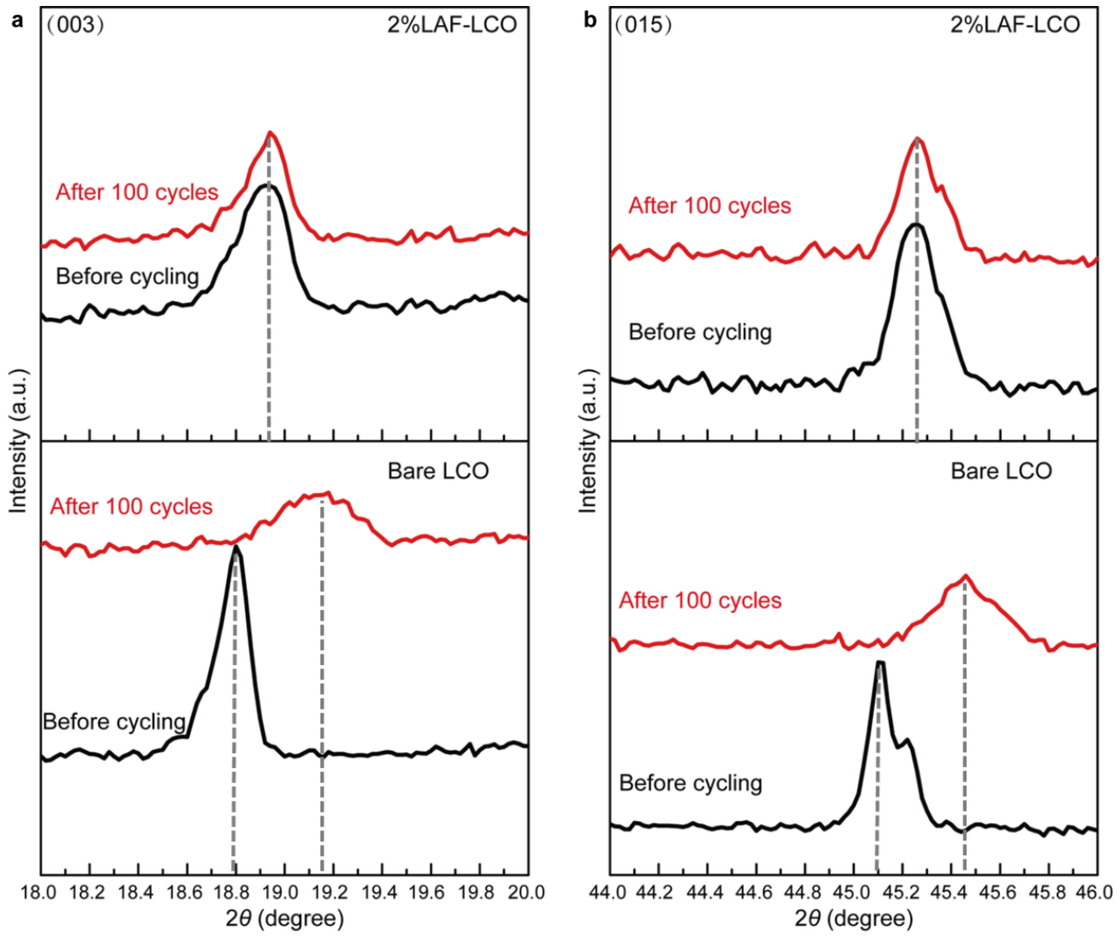

Supplementary Figure 14 | The (003) and (015) peaks evolution of bare LCO or 2% LAF-LCO before cycling and after 100 cycles at 137 mA g^{-1}. Source data are provided as a Source Data file.

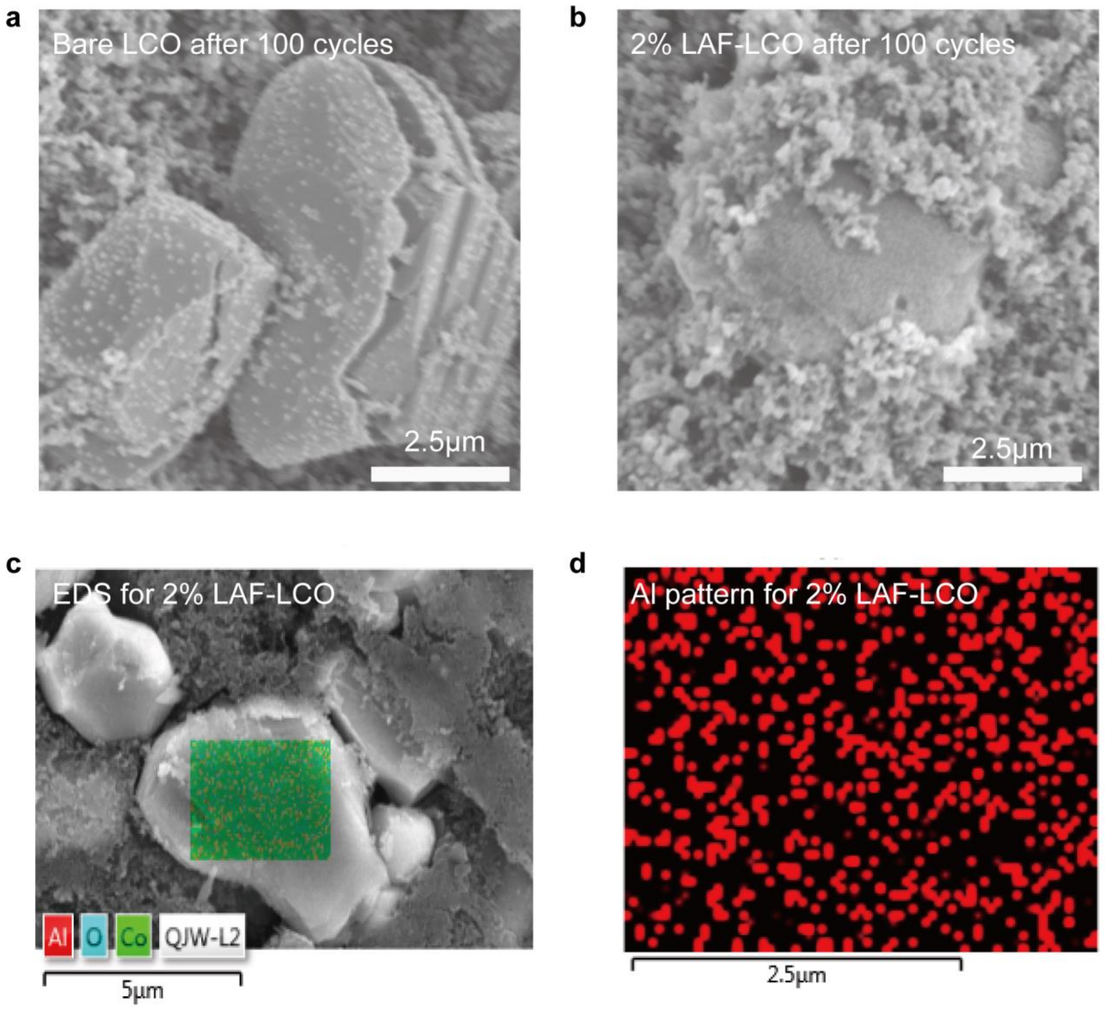

Supplementary Figure 15 | The detailed morphology analyses of bare LCO and 2% LAF-LCO electrodes. a-b SEM images of bare LCO and 2% LAF-LCO electrodes after 100 cycles at room temperature in the voltage range of 3.0-4.6 V (vs. Li^+/Li) at 137 mA g^{-1}; **c-d** The EDS elemental maps of 2% LAF-LCO electrodes after 100 cycles.

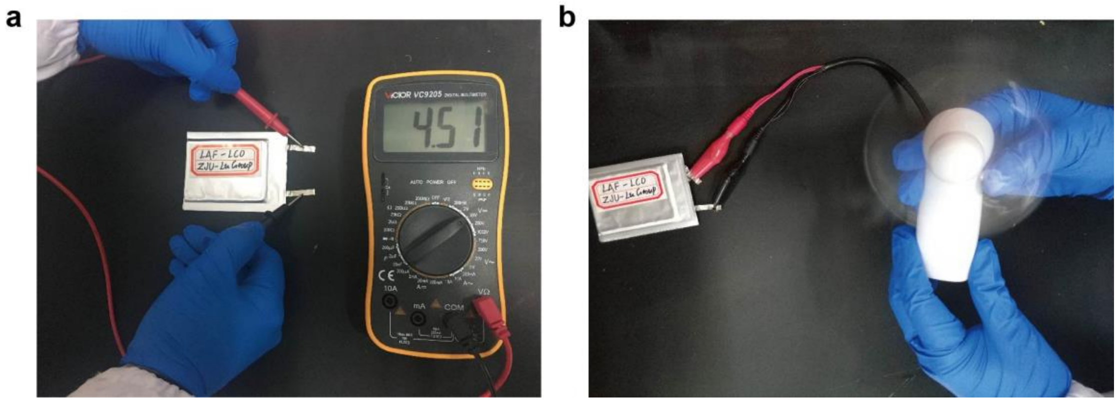

Supplementary Figure 16 | The practical application of graphite/2%LAF-LCO full-cells. a Open-circuit voltage of pouch-type full-cells with 2% LAF-LCO electrode after SEI formation and first charge process. **b** Hand-held electric fan, which usually uses three triple-A cells with working voltage of 4.5 V, was successfully operated by using the pouch-type full-cell with 2% LAF-LCO electrode.

Supplementary Table 1 | Comparison of electrochemical performance from other research strategies of LCO surface coating at high cut-off voltage of 4.6 V.

Coating type/mate1rial	Electrochemical performance (cycling at 4.6V)	Ref.
Al_2O_3-LCO derived by $Al_2(SO_4)_3$	155 mAh g^{-1},100 cycles, 140mA g^{-1}, 0.224 mA cm^{-2}	1
AlF_3 coated LCO	172 mAh g^{-1}, 50 cycles, 80 mA g^{-1}	2
CeF_3 coated LCO	160 mAh g^{-1}, 27 cycles, 40 mA g^{-1}	3
Li_2CO_3 coated LCO	133 mAh g^{-1}, 50 cycles, 28 mA g^{-1}	4
Li_2MnO_3 coated LCO	102 mAh g^{-1}, 100 cycles, 155 mA g^{-1}	5
$LiAlO_2$ coated LCO by ALD	172.2 mAh g^{-1}, 50 cycles, 20 mA g^{-1}, 0.300 mA cm^{-2}	6
Mg doping and ZrO_xF_y coated LCO	136.6 mAh g^{-1}, 100 cycles, 155 mA g^{-1}	7
Hydrothermal assisted LAF-LCO	170.7 mAh g^{-1}, 200 cycles, 27.4 mA g^{-1}, 0.276 mA cm^{-2}<br>158.8 mAh g^{-1}, 100 cycles, 137 mA g^{-1}, 1.38 mA cm^{-2}	This work

Supplementary Note 1 | The detailed calculation process for Stoichiometry predictions of Li-Al-Co-O-F solid solution

We first considered the following reaction:

$$\mathrm{Li{+}CoO_2 \rightarrow LiCoO_2} \quad (1)$$

which we determined as exoergic by -3.69 eV showing the stability of $LiCoO_2$. Under an electric field of 4.6 V, the concentration of Li is reduced to form $Li_{0.13}CoO_2$[8]. We used a $Li_{1/9}CoO_2$ supercell to simulate the crystal structure with this stoichiometry. Supplementary Fig. 2a illustrates the atomic structure. We then compared the formation energy of the following reaction:

$$\frac{1}{9}\mathrm{Li{+}CoO_2 \rightarrow Li_{1/9}CoO_2} \quad (2)$$

We found that Reaction (2) is also exoergic whereas the formation energy (-0.06 eV) is much smaller, implying that $Li_{1/9}CoO_2$ is less stable, which is in agreement with the experimental phenomenon.

Electric field also has significant effects on the stability of Li-Al-F compound. First, Li-Al-F compound is predicted to decompose into Li, F_2 and AlF_3 at a high potential of 5.72 V. Second, Li-Al-F compound interacts with Li to produce Li_3AlF_6 and Al at a low potential of 2.05 V[9]. We expect that, at an intermediate potential (4.6 V) as used in our experiment, both reactions could occur, leading to excessive Li, Al and F that are soluble in $LiCoO_2$ to form a solid solution of Li-Al-Co-O-F (Al occupying the site of Co, F occupying the site of O). To determine the approximate stoichiometry of this solution, we used the cluster expansion method to determine the optimal concentration x of Al in $LiAl_xCo_{1-x}O_2$ using the parent $LiCoO_2$ structure. Supplementary Fig. 3 shows the formation energy of $LiAl_xCo_{1-x}O_2$ with different concentrations. We found that

the energy phase diagram exhibits a global minimum at the concentration of 1/3. Namely, the $LiAl_{1/3}Co_{2/3}O_2$ compound shows the lowest energy. The structure of this compound is displayed in Supplementary Fig. 2b. Since there is no available crystal structure data for a quinary Li-Al-Co-O-F compound, we applied the special quasirandom structures (SQS) method[10] to generate a supercell that ensure the ratio between the concentrations of O and F to be 1/3. For balancing the charge, the representative Li-Al-Co-O-F compound is defined as $Li_{1/3}Al_{1/3}Co_{2/3}O_{4/3}F_{2/3}$ based on the following possible reactions:

$$\frac{1}{3}\text{Li}+\frac{2}{3}\text{CoO}_2+\frac{1}{3}\text{Al}+\frac{1}{3}\text{F}_2 \rightarrow \text{Li}_{1/3}\text{Al}_{1/3}\text{Co}_{2/3}\text{O}_{4/3}\text{F}_{2/3} \quad (3)$$

and

$$\frac{1}{9}\text{Li}+\frac{2}{3}\text{CoO}_2+\frac{1}{3}\text{Al}+\frac{1}{3}\text{F}_2 \rightarrow \text{Li}_{1/9}\text{Al}_{1/3}\text{Co}_{2/3}\text{O}_{4/3}\text{F}_{2/3} \quad (4)$$

We obtained a $Li_{1/3}Al_{1/3}Co_{2/3}O_{4/3}F_{2/3}$ structure that is shown in Supplementary Fig. 2c. We computed the energy change of Reaction (3) and found that this reaction is more strongly exoergic than Reaction (1) (-4.48 vs. -3.69) eV. Due to no available evidence for the deintercalation of Li ion from $Li_{1/3}Al_{1/3}Co_{2/3}O_{4/3}F_{2/3}$ at 4.6 V, we propose a hypothesis that $Li_{1/9}Al_{1/3}Co_{2/3}O_{4/3}F_{2/3}$ may be formed, having the same amount of Li with $Li_{1/9}CoO_2$. We also calculated the formation energy of $Li_{1/9}Al_{1/3}Co_{2/3}O_{4/3}F_{2/3}$ in Reaction (4) with the structure illustrated in Supplementary Fig. 2d. Similarly, the formation energy became more exoergic changing from -0.06 of $Li_{1/9}CoO_2$ to -3.56 eV. The *c*-lattice parameter of $LiCoO_2$ (14.35 Å), $Li_{1/9}CoO_2$ (13.98 Å), $Li_{1/3}Al_{1/3}Co_{2/3}O_{4/3}F_{2/3}$ (14.80Å), and $Li_{1/9}Al_{1/3}Co_{2/3}O_{4/3}F_{2/3}$ (14.60 Å) were obtained via above methods.

Supplementary references

1. Zhou, A. *et al.* Al_2O_3 surface coating on $LiCoO_2$ through a facile and scalable wet-chemical method towards high-energy cathode materials withstanding high cutoff voltages. *J. Mater. Chem. A* **5**, 24361–24370 (2017).
2. Sun, Y. K., Chong, S. Y., Myung, S. T., Belharouak, I. & Amine, K. Role of AlF_3 coating on $LiCoO_2$ particles during cycling to cutoff voltage above 4.5 V. *J. Electrochem. Soc.* **156**, A1005–A1010 (2009).
3. Aboulaich, A. *et al.* Improving thermal and electrochemical performances of $LiCoO_2$ cathode at high cut-off charge potentials by MF_3 (M=Ce, Al) coating. *Mater. Res. Bull.* **73**, 362–368 (2016).
4. Dai, X. *et al.* Extending the high-voltage capacity of $LiCoO_2$ cathode by direct coating of the composite electrode with Li_2CO_3 via magnetron sputtering. *J. Phys. Chem. C* **120**, 422–430 (2015).
5. Wang, Z., Wang, Z., Guo, H., Peng, W. & Li, X. Synthesis of Li_2MnO_3-stabilized $LiCoO_2$ cathode material by spray-drying method and its high-voltage performance. *J. Alloys Compd.* **626**, 228–233 (2015).
6. Xie, J. *et al.* Engineering the surface of LiCoO 2 electrodes using atomic layer deposition for stable high-voltage lithium ion batteries. *Nano Res.* **10**, 1–11 (2017).
7. Wang, Z. *et al.* Mg doping and zirconium oxyfluoride coating co-modification to enhance the high-voltage performance of $LiCoO_2$ for lithium ion battery. *J. Alloys Compd.* **621**, 212–219 (2015).
8. Radin, M. D. *et al.* Narrowing the gap between theoretical and practical capacities in Li-ion

layered oxide cathode materials. *Adv. Energy Mater.* **7**, 1602888 (2017).

9. Xie, J. *et al.* Atomic layer deposition of stable $LiAlF_4$ lithium ion conductive interfacial layer for stable cathode cycling. *ACS Nano* **11**, 7019–7027 (2017).

10. Zunger, A., Wei, S., Ferreira, L. G. & Bernard, J. E. Special quasirandom structures. *Phys. Rev. Lett.* **65**, 353–356 (1990).
